# The Pattern of microRNA Binding Site Distribution

**DOI:** 10.3390/genes8110296

**Published:** 2017-10-27

**Authors:** Fangyuan Zhang, Degeng Wang

**Affiliations:** 1 Department of Mathematics and Statistics, Texas Tech University, Lubbock, TX 79409, USA; 2 Department of Environmental Toxicology, The Institute of Environmental and Human Health (TIEHH), Texas Tech University, 1207 Gilbert Dr., Lubbock, TX 79416, USA

**Keywords:** microRNA, small-world network, scale-free network, clustering coefficient, distribution

## Abstract

Micro-RNA (miRNA or miR) regulates at least 60% of the genes in the human genome through their target sites at mRNA 3’-untranslated regions (UTR), and defects in miRNA expression regulation and target sites are frequently observed in cancers. We report here a systematic analysis of the distribution of miRNA target sites. Using the evolutionarily conserved miRNA binding sites in the TargetScan database (release 7.1), we constructed a miRNA co-regulation network by connecting genes sharing common miRNA target sites. The network possesses characteristics of the ubiquitous small-world network. Non-hub genes in the network—those sharing miRNA target sites with small numbers of genes—tend to form small cliques with their neighboring genes, while hub genes exhibit high levels of promiscuousness in their neighboring genes. Additionally, miRNA target site distribution is extremely uneven. Among the miRNAs, the distribution concentrates on a small number of miRNAs, in that their target sites occur in an extraordinarily large number of genes, that is, they have large numbers of target genes. The distribution across the genes follows a similar pattern; the mRNAs of a small proportion of the genes contain extraordinarily large numbers of miRNA binding sites. Quantitatively, the patterns fit into the P_(K)_ ∝ K^−α^ relationship (P_(K)_: the number of miRNAs with K target genes or genes with K miRNA sites; α: a positive constant), the mathematical description of connection distribution among the nodes and a defining characteristic of the so-called scale-free networks—a subset of small-world networks. Notably, well-known tumor-suppressive miRNAs (Let-7, miR-15/16, 26, 29, 31, 34, 145, 200, 203–205, 223, and 375) collectively have more than expected target genes, and well-known cancer genes contain more than expected miRNA binding sites. In summary, miRNA target site distribution exhibits characteristics of the small-world network. The potential to use this pattern to better understand miRNA function and their oncological roles is discussed.

## 1. Introduction

It is now clear that microRNA (miRNA or miR) aberrancy is a critical factor in cancer. Oncogenic genetic alterations are responsible for cancer initiation, gradual enlargement and disorganization of tumor tissues and, ultimately, metastasis [1]. For a long time, alterations in oncogene and tumor-suppressor gene coding regions were considered to be the only causes of tumorigenesis, as these genes are involved in cellular pathways underneath key physiological processes such as cell cycle, apoptosis and cellular homeostasis. Recent studies have shown that this can occur in both coding and non-coding genomic regions. Many studies have identified a large number of non-coding RNA (ncRNA) transcripts with no significant open reading frame. Yet these transcripts are involved in key biological processes, such as cell cycle, and exhibit aberrancy in cancers [2]. The miRNA, a family of approximately 20–22 nucleotide long RNAs, is a prominent category of ncRNA [3,4,5].

MiRNA was initially discovered in 1993 in *Caenorhabditis elegans* [6]. Subsequently, it became clear that miRNA is a conserved regulatory mechanism that has broad functional significance throughout the plant and animal kingdoms. The biogenesis of microRNAs has been, as discussed below, well characterized and is evolutionally conserved [7]. The miRNAs reside in various genomic contexts. They can be found in both intronic and intergenic regions; a miRNA gene may encode a single microRNA hairpin precursor, or a cluster of multiple precursors. The primary transcript produced by RNA polymerases II and III is cleaved in the nucleus into the precursor hairpin. There are currently over 28,000 miRNA precursor hairpins, 1881 of which are human, collected in the miRBase database of miRNAs and their annotation [8]. The precursor hairpin is exported into the cytoplasm. Mature miRNA is then produced from it by the Dicer complex and loaded onto the RNA-induced silencing complex (RISC). The mature single-stranded miRNA, associated with the Argonaute 2 (AGO2) protein in the RISC complex, typically binds to the 3’-untranslated regions (UTRs) of target messenger-RNAs (mRNAs) [9]. The consequence can be reduced translation, enhanced degradation of the target mRNAs, or both. Initially, miRNAs were thought to function primarily to negatively regulate translation, with the fate of the mRNA depending on the degree of base-pairing complementarity between the mRNA molecule and the “seed” region at the 5′-end of the miRNA [10]. Recent studies have, however, given mRNA destabilization a more prominent role, as target mRNA degradation preceded translation inhibition upon ectopic miRNA expression through expression vectors in these studies [11,12].

Oncological miRNA aberrancy has been well documented [13]. One type of aberrancy is miRNA biogenesis and expression changes [14]. Global reduction of miRNA expression in cancer is frequently observed [15,16]. A small number of miRNAs stand out. For example, miR-15 and miR-16 expression is often completely abolished due to their location in chromosome 13q14.3—a region frequently deleted in chronic lymphocytic leukemia [17]. As well, some miRNAs are oncogenic and often overexpressed; for example, the miR-21 in breast cancer, glioblastoma, head and neck cancer, ovarian cancer, B-cell lymphoma, hepatocellular carcinoma, cervical cancer, and lung cancer [18,19,20,21,22,23]. Another type of aberrancy is mutation of miRNA binding sites in the 3’-UTRs, rendering the corresponding mRNAs insensitive to miRNA regulation [24,25,26,27,28].

A key, and perhaps the biggest, challenge in miRNA research is the complexity of the miRNA-mRNA target relationship. This is incurred by the short length of miRNA binding site, typically six to eight bases in human. Each miRNA has the potential to target a huge number of mRNAs, and one mRNA species can be regulated by multiple miRNAs. Additionally, the short length of miRNA binding site renders sequence-based miRNA target prediction impractical due to high noise levels. This has hindered a thorough understanding of miRNA actions in normal and oncological cellular processes.

Thus, in this initial study, we tried to tackle the complexity of miRNA-target relationship. Instead of focusing on individual miRNAs and their target, we studied genomic distribution of the whole set of evolutionarily conserved miRNA target sites to uncover fundamental patterns and principles. We report that miRNA co-regulation network, in which genes are connected by shared miRNA target sites in their mRNAs, possesses small-world network characteristics. Well-known cancer genes contain more than expected miRNA target sites in their mRNAs, and tumor suppressive miRNAs target more than expected mRNAs.

## 2. Materials and Methods 

### 2.1. Evolutionarily-Conserved miRNA Binding Sites

To alleviate the high noise issue associated with miRNA binding site prediction, we restrict our analysis to evolutionarily conserved human miRNA binding sites. The set of sites were downloaded from the TargetScan database 7.1 (June 2016 release) in June 2017 [29]. At the time of download, this was the most current version. The dataset contains 116,371 miRNA binding sites in the 3’-UTRs of 12,455 human genes.

### 2.2. MiRTarBase Data

We downloaded the miRTarBase release 7.0 (September 2017 release) human data from its websites [30]. At the time of download, this was the most current version. The data was used as a list of experimentally determined miRNA-mRNA target relationship.

### 2.3. Identification of Cancer Genes

For this analysis, our priority is to use a list of high-confidence cancer genes, not the comprehensiveness of the list. Thus, we used a list of 125 cancer driver genes identified by Wood et al. through rigorous analysis [31]. The list includes both oncogenes and tumor suppressor genes, and covers a large number of key cancer pathways. While a much larger number of mutations can be identified in tumor tissues, a big portion of the mutations tends to be passenger, instead of driver, mutations. The genes and their annotation are listed in supplemental Table S1.

### 2.4. Well-Known Tumor Suppressive miRNAs

We collected well-known tumor suppressive miRNAs to exemplify the global reduction of miRNA expression in cancer. We used the well-known tumor suppressive miRNAs identified in Hayes et al [13]. We also included the miRNAs listed in Garzon et al. [32] and Blandino et al. [33]. A total of 13 miRNAs are included. They are Let-7, miR-15/16, 26, 29, 31, 34, 145, 200, 203–205, 223, and 375.

### 2.5. Computer Software

The open source software package R (version 3.1) was used for most analysis. Practical Extraction and Reporting Language (PERL) was used to compute clustering coefficient in the miRNA co-regulation network.

### 2.6. Clustering Coefficient

We used the standard procedure to calculate clustering coefficient for each gene [34,35]. Briefly, all immediate neighbors of a gene in the network were collected. The clustering coefficient (*C_i_*) of the gene was calculated as the proportion of gene pairs among its neighbors that are mutually connected, with following formula:

*C_i_* = 2*N_i_*/*K_i_*(*K_i_* − 1). (1)

*K_i_*: immediate neighbor count of gene *i*; *N_i_*: pairwise connection count among immediate neighbors of gene *i*.

## 3. Results

### 3.1. Evolutionarily Conserved miRNA Binding Sites and miRNA Co-Regulation Network

Despite the well-documented significance of miRNA-mediated regulation in many cellular processes, miRNA target site distribution has not been fully explored to uncover fundamental organizational principles. Our goal is to contribute to a satisfactory solution of this issue. We adopted the approach of utilizing evolution conservation to reduce the high levels of noise in genomic sequence analysis, a particularly troublesome technical challenge of computational analysis of miRNA-mRNA target relationship. Fortunately, the TargetScan database already accomplished this task. We therefore downloaded the evolutionarily conserved miRNA binding sites from this database (see Materials and Methods) [29]. While this dataset only covers the 3’-UTR, the limitation is partially offset by the fact that the majority of the miRNA binding sites are in the 3’-UTRs. To the best of our knowledge, this is the best possible, though not perfect, option to perform unbiased large-scale analysis of miRNA binding sites.

To provide a platform for our systematic analysis, we constructed a miRNA co-regulation network of the 12,455 human genes in the dataset. When a pair of genes share at least one common miRNA binding sites in their 3’-UTRs, we created an undirected connection between them in the network; hence, the name miRNA co-regulation network. A total of 43,027,373 connections were created among the 12,455 genes.

### 3.2. The Co-Regulation Network Possesses Small-World Network Characteristics

**Figure 1:**
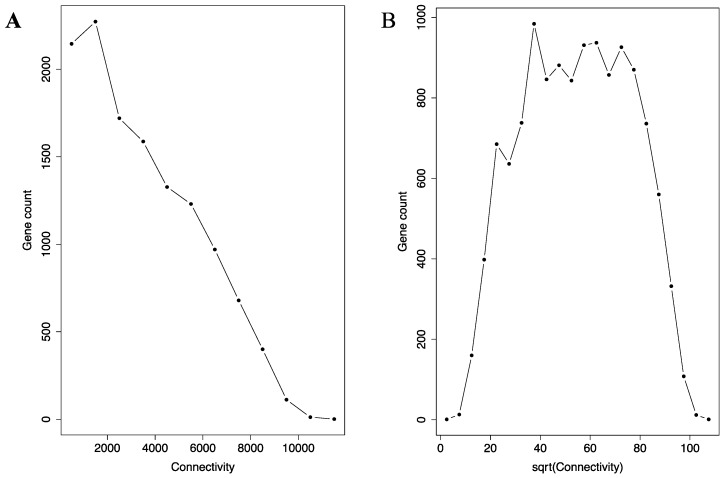
The connectivity distribution in the micro-RNA (miRNA) co-regulation network is similar to a square root distribution. The histogram of connectivity (**A**) and the square root (sqrt) of the connectivity (**B**) are shown. The connectivity histogram (**A**) is heavily right skewed, with a small portion of the genes having more than 10,000 connections and a much bigger portion with fewer than 2000 connections. Upon square root transformation, the histogram (**B**) becomes bell-shaped and approximately symmetrical.

Construction of the network enabled us to utilize key standard network analysis parameters and methods. First, we examined the connection distribution among the genes. Network analysis has revealed that many real-world networks belong to small-world network. In such networks, the connection is not evenly distributed. Instead, they concentrate on a small portion of hub (or, celebratory) nodes in the network. In a subset of small-world network called scale-free network, the distribution can be described by the P_(K)_ ∝ K^−α^ relationship (P_(K)_: the number of nodes with K immediate neighbors in the network; α: a positive constant). As shown by the histogram in Figure 1A, the connection distribution is indeed highly right skewed, which means the distribution focuses heavily on a small number of hub genes in the miRNA co-regulation network. A very small portion of the genes have more than 10,000 connections, while a much bigger portion having fewer than 2000 connections. However, the network is not scale-free. Instead, the histogram in Figure 1A is likely a square root distribution, as square root transformation transformed the histogram into a roughly symmetrical bell-shaped distribution (Figure 1B). Any distribution following the P_(K)_ ∝ K^−α^ relationship will not transform to such symmetric bell-shaped distribution.

### 3.3. Negative Correlation between Connectivity and Clustering Coefficient in the Co-Regulation Network

Another commonly used network analysis parameter is clustering coefficient. It quantifies the heterogeneity of the immediate neighbors of a node in the network; the lower the heterogeneity, the higher the clustering coefficient. Mathematically, the clustering coefficient of a node is the proportion of the pairs among its neighbors that are also mutually connected. A high clustering coefficient value (and, thus, low heterogeneity) means the node and its neighbors form a close clique. In the small-world network, the trend is that the heterogeneity increases when the connectivity of the node increases. That is, low connectivity nodes tend to form close cliques with their respective immediate neighbors. 

**Figure 2:**
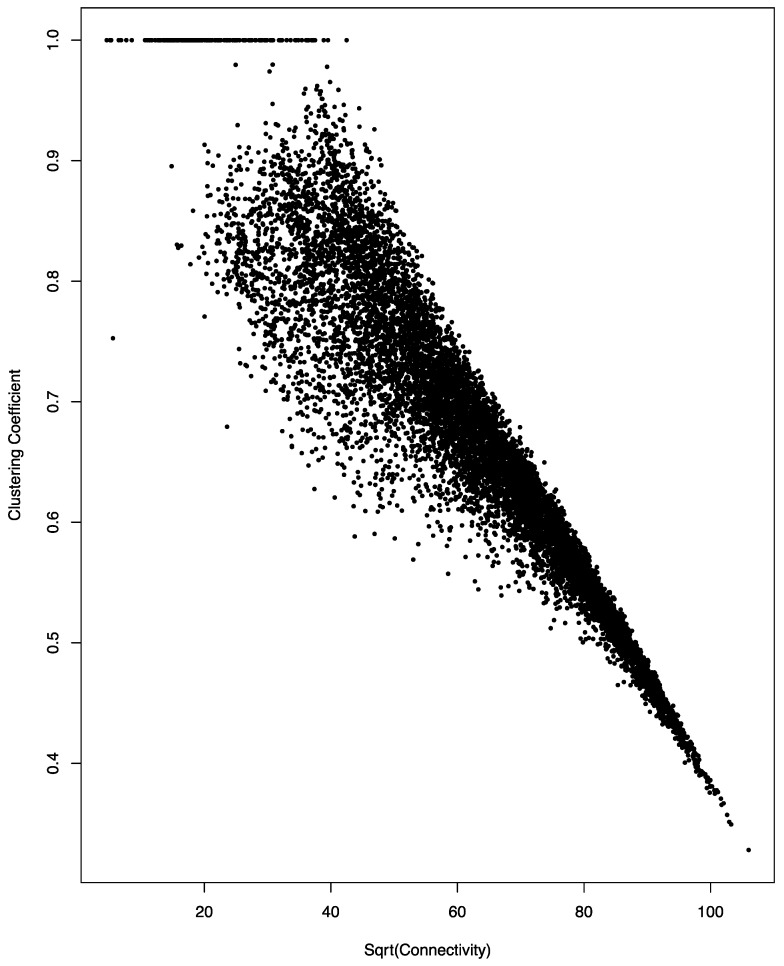
Negative correlation between connectivity and clustering coefficient in the miRNA co-regulation network. A scatter plot of the square root (sqrt) of connectivity and clustering coefficient is shown. The linear relationship has a correlation coefficient of 0.94.

As shown in Figure 2, this is indeed the case for the miRNA co-regulation network. An almost perfect negative linear relationship was observed between the square root of connectivity and the clustering coefficient of the genes in the co-regulation network; the correlation coefficient of the two parameters is 0.94.

### 3.4. High Connectivity of Cancer Genes in the Co-Regulation Network

**Figure 3:**
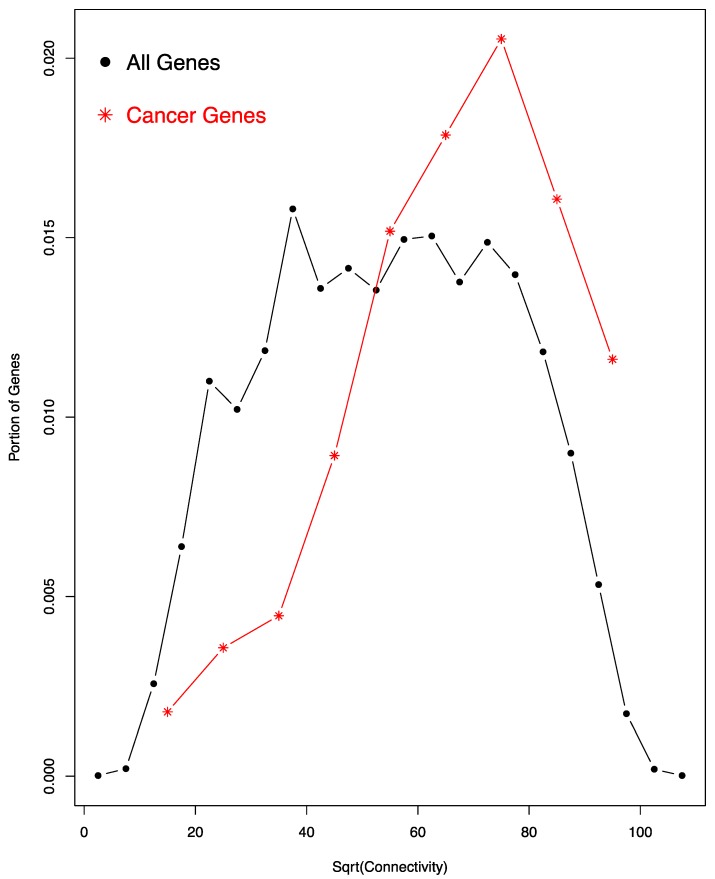
Cancer genes have higher connectivity in the network. A comparison of the histograms of the square root (sqrt) of all genes (black dots and lines) and cancer genes (red stars and lines) is shown. The histogram of cancer genes exhibits a significant right shift. According to bootstrapping, the difference in the connectivity of the two groups has a *p*-value smaller than 0.001 (see text for detail).

We also examined whether cancer genes exhibit special characteristic in the miRNA co-regulation network. To accomplish this, we used a previously identified set of 125 cancer driver genes, both oncogenes and tumor suppressor genes [31]. Of these, 112 (89.6%) are also included in the miRNA co-regulation network; the percentage is much higher than the genome-wide percentage (~60%) of genes regulated by miRNAs. As shown in Figure 3, these 112 cancer genes exhibit a much higher level of connectivity than expected, in that its histogram exhibits a significant right shift towards high connectivity range. 

To calculate the significance of the increase of tumor suppressor gene connectivity in the co-regulation network by bootstrapping, we randomly sampled 112 genes from the whole gene set and calculated their mean connectivity. We repeated the process 1000 times, generating 1000 mean connectivity values, which follow a normal distribution. None of the 1000 mean values is equal to or bigger than the observed mean connectivity of the 112 cancer genes. Thus, the *p*-value for the increase of cancer gene connectivity must be smaller than 0.001 (1/1000). A Student’s *t*-test of the 1000 values and the observed mean connectivity of the 112 cancer genes resulted in a *p*-value smaller than 2.2 × 10^−16^. Thus, cancer genes exhibit much higher than expected connectivity.

### 3.5. Distribution of Target Genes among the miRNAs

**Figure 4:**
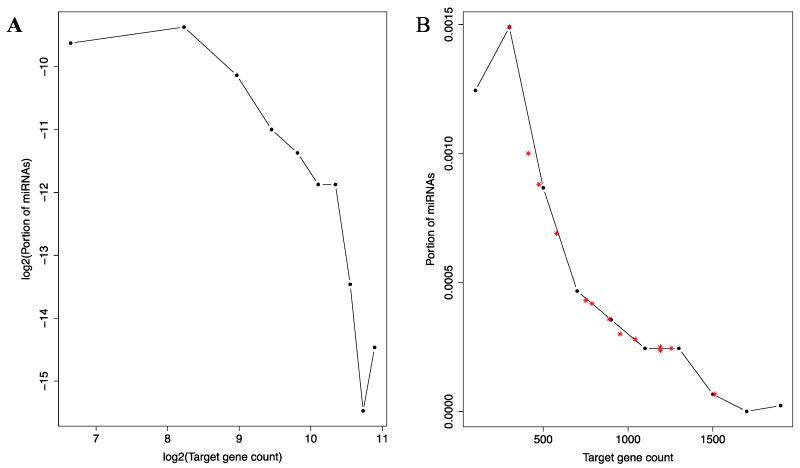
Histogram of miRNA binding site distribution across the miRNAs. A log-log plot (**A**) and an un-logged plot (**B**) of the histogram is shown. In A, the curve depicts a negative correlate that resembles previously described log-log histograms of many types of scale-free networks [36]. In B, the approximate location of five well-known tumor suppressive miRNAs are marked with red asterisks symbols to illustrate their higher than expected target gene counts.

Given the small-world characteristics of the co-regulation network, we also directly examined the distribution of the 12,455 target genes across the miRNAs. As shown in Figure 4A, this distribution is also heavily concentrated, even more so than the connectivity distribution in the co-regulation network. The distribution closely resembles the P_(K)_ ∝ K^−α^ relationship (P_(K)_: the number of miRNAs with K target genes; α: a positive constant) previously described for many types of networks [36]. This relationship describes, as discussed earlier, the connectivity distribution and a defining characteristic of the scale-free network, a subset of the small-world networks [36]. It will be interesting to investigate which aspects of miRNA regulatory functions give rise to this characteristic.

Notably, well-known tumor suppressive miRNAs exhibit more than expected target genes (Figure 4B). The 13 well-known tumor suppressive miRNAs (see Materials and Methods) are used in this analysis. Their target gene counts are contrasted with the histogram of target gene counts of the whole miRNA set. As shown in Figure 4B, all but one miRNAs are located to be right of the peak of the histogram. Thus, they target much more genes than expected by random chance from the overall histogram.

### 3.6. Distribution of miRNA Binding Sites among the Genes

**Figure 5:**
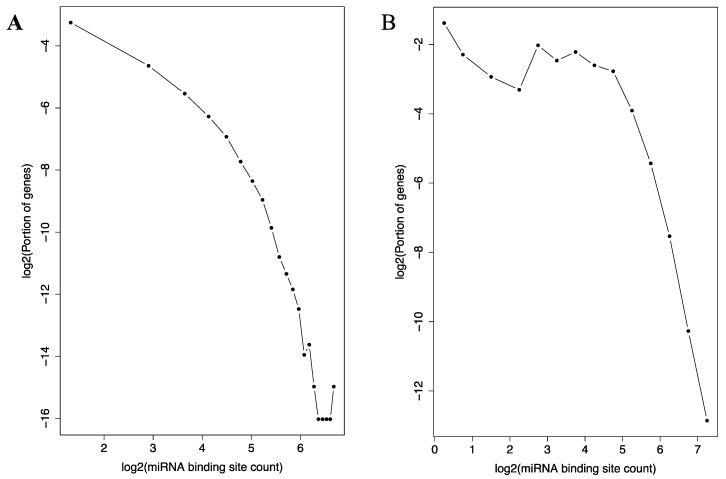
Histogram of miRNA binding site distribution across the genes. Log-log plots of the histograms are shown for the TargetScan (**A**) and the miRTarBase (**B**) datasets. The curve in A depicts a negative correlation that resembles previously described log-log histograms of many types of scale-free networks [36]. The same is observed in B at high connectivity range.

Similarly, the distribution of the 116,371 miRNA binding sites among the genes also follow the relationship (Figure 5A). A small number of genes possesses extremely high numbers (more than 60) of binding sites in their 3’-UTRs, while the majority of the genes only a few of the sites. Interestingly, AGO1, AGO2 and AGO3—three import proteins in miRNA function—are all ranked in the top 15 among all the genes in term of the miRNA binding site count in their 3-UTRs.

Additionally, in experimentally determined miRNA-mRNA target relationships collected in the miRTarBase database, similar pattern was observed (Figure 5B). The majority of the genes are bound to by a small number of miRNAs, while a small number of genes are bound to by more than 60. As in the TargetScan dataset, AGO1, AGO2 and AGO3 are all highly ranked among all the genes in terms of the counts of miRNAs that bind to their mRNAs.

We also tested, with bootstrapping, whether the cancer genes possess more than expected miRNA binding sites in their 3’-UTRs. We once again randomly picked 112 genes and calculated their mean binding site count. This process was repeated 1000 times, generating 1000 values. The 1000 values have a mean of 8.4, and a standard deviation of 0.86. We then compare these values with the mean binding site count of the 112 cancer genes, which is 14. None of the 1000 randomly generated mean values is equal to or bigger than 14. Thus, the *p*-value of the difference between the expected value and the observed values is definitely smaller than 0.001 (1/1000). A Student’s *t*-test of the randomly generated mean values and the observed mean value gave a significant *p*-value smaller than 2.2 × 10^−16^. Thus, the cancer driver genes possess much more than expected miRNA binding sites.

## 4. Discussion

It is widely accepted that miRNAs play important regulatory roles in many cellular processes, and their aberrancy contributes significantly to tumorigenesis. However, a thorough understanding of miRNA function is currently being hindered by our lack of reliable approaches to systematically identify and study miRNA-mRNA target relationship. It is our hope that this initial study will contribute to some progress in this aspect.

One source of difficulty in studying miRNA-mRNA target relationship is the complexity of the relationship. Each individual miRNA can potentially target a high number of mRNAs; and each mRNA can potentially possess a large number of miRNA binding sites in its 3’-UTR. This one-to-many relationship is not unique to miRNA. It is similar to transcription regulation, where one transcription factor can regulate many genes and one gene can be regulated by multiple transcription factors. This complexity has been a tremendous challenge in studying human transcription regulation. Likewise, it is now obvious that the complexity is a challenge in studying miRNA regulatory activity as well. 

Even though the one-to-many relationship has long been known, it has not been quantitatively studied. Utilizing the miRNA co-regulation network as a platform, we were able to analyze the miRNA-mRNA target relationship in a quantitative manner. The results show that the miRNA co-regulation network we constructed belong to the small-world network, in terms of connectivity distribution and clustering coefficient values of the genes. Thus, the small-world network concept should be a useful guiding framework for further systematic analysis of the complexity of miRNA regulatory function, enabling the utilization of the suit of network parameters and analysis approaches that have been fruitfully used in a wide range of research domains. 

Our results also showed that features of the scale-free network, a subset of small-world networks, should also apply to miRNA functional studies. The distribution of miRNA binding sites follows the P_(K)_ ∝ K^−α^ relationship, a defining characteristic of scale-free network [36]. This is true when the distribution is analyzed across either the miRNAs or the mRNAs. However, the miRNA co-regulation network we constructed in this study does not share this feature; its connectivity distribution follows, instead, the square root distribution. Thus, we will need to either modify our network model or construct new relevant models, in order to take full advantages of this scale-free feature in our future studies. 

MiRNA regulation shares another similarity with transcription regulation: the incredibly fuzzy or weak signal individual bind site gives out. Short miRNA binding sites has been a frustrating challenge in computational analysis of miRNA function. Similarly, human transcription factor binding sites are notoriously short, making sequence-based binding site prediction incredibly noisy [37]. This challenge of short transcription factor binding sites was partially alleviated by utilization of genomic context information, such as chromatin density and epigenetic modification. Additionally, the challenge is partially alleviated by utilizing combinatorial patterns of multiple transcription factor binding sites to increase the signal-to-noise ratio [38], as well as by identification of evolutionarily conserved sites [39]. As discussed earlier, evolution conservation is already fruitfully applied to miRNA study [29]. It will be interesting to see whether the combinatorial pattern of multiple sites can also be applied to improve the power of computational analysis in systematic miRNA functional studies.

Our analysis also showed that key cancer genes and tumor suppressive miRNAs hold a prominent status in miRNA regulation network. The former possesses higher connectivity in the miRNA co-regulation network and more miRNA binding sites in their 3’-UTRs. The latter has more than expected target genes. This is not surprising, as these genes and miRNAs must be controller of key cellular processes and thus must be abrogated in order for cancer to initiate and develop. Hopefully, our network-based miRNA analysis will provide a new way to characterize these crucial genes and miRNAs. It is critical to identify, and/or develop new software to traverse the small-world network pattern and uncover additional mechanistic insights.

In summary, this study introduced the network analysis into miRNA functional study. We hope this will enable the utilization of the set of network parameters and analysis approaches to catalyze the advancement of a systematic understanding of miRNA functions. This will be crucial for a thorough understanding of gene expression complexity such as the discrepancy among key gene expression parameters [40,41,42].

## Supplementary Materials

The following are available online at www.mdpi.com/2073-4425/8/11/296/s1. Table S1: List of cancer genes used in this study and their annotation.
